# The effect of iatrogenic subclinical hyperthyroidism on anxiety, depression and quality of life in differentiated thyroid carcinoma

**DOI:** 10.3906/sag-1902-176

**Published:** 2020-06-23

**Authors:** Nazlı GÜLSOY KIRNAP, Özlem TURHAN İYİDİR, Yusuf BOZKUŞ, Şerife Mehlika IŞILDAK, Cüneyd ANIL, Sevde Nur FIRAT, Canan DEMİR, Aslı NAR, Neslihan BAŞÇIL TÜTÜNCÜ

**Affiliations:** 1 Department of Endocrinology and Metabolism, Faculty of Medicine, Başkent University, Ankara Turkey

**Keywords:** Beck anxiety and depression inventory, differentiated thyroid carcinoma, SF-36 quality of life survey, subclinical hyperthyroidism, TSH suppression

## Abstract

**Background/aim:**

Overt thyroidism is known to cause neuropsychiatric disorders but studies on subclinical hyperthyroidism (SCH) are limited. Subclinical hyperthyroidism induction by administering L-Thyroxine (LT4) is the standard treatment method in differentiated thyroid carcinoma (DTC) follow-up. Our aim was to investigate whether anxiety, depression and quality of life are affected in DTC patients followed-up with exogenous SCH.

**Materials and methods:**

The patients were divided into exogenous SCH by LT4-DTC (n = 127), euthyroid-DTC (n = 66) and exogenous euthyroid-benign thyroid noduüle (BTN) who underwent thyroidectomy for benign thyroid pathology (n = 85) groups.

**Results:**

The rate of moderate/severe anxiety was significantly higher in SCH-DTC than euthyroid-BTN group (27.5%, n = 35 vs. 9.4%, n = 8) (P = 0.001). TSH levels and Beck anxiety inventory (BAI) scores were significantly negatively correlated(P = 0.009 r = –0.16). Free T4 and BAI were significantly positively correlated (P = 0.04 r = 0.4). The groups were similar in terms of depression severity (P = 0.15). Subclinical hyperthyroid-DTC group scored significantly lowerthan euthyroid-BTN group in all scales of SF-36 quality of life survey.

**Conclusion:**

LT4-induced SCH, which is a part of traditional DTC treatment, can exacerbate the anxiety symptoms in patients and disrupt their quality of life, depending on the level of fT4.

## 1. Introduction

Differentiated thyroid carcinoma is the most common type of endocrinological neoplasm [1]. The disease has a general mortality rate of less than 10%, and most patients have a long survival due to the tumour’s biological behaviour and treatment’s efficacy [2]. Conventional treatment for DTC includes total or partial thyroidectomy, remnant tissue radioactive iodine (RAI) ablation, and thyroid stimulating hormone/thyrotropin (TSH) suppression with LT4. The latter serves to create an exogenous SCH (a normal serum free T3 (fT3) free T4 (fT4) but with a reduced TSH concentration) [3]. These chemical changes are associated with increased cardiovascular risk, fatigue, reduced bone mineral density, and impaired muscle function [4–7]. Such changes may affect patients’ physical and psychological status and impair their quality of life (QoL) [7,8] and they may also be associated with depression [9,10] and anxiety [11,12]. Affect and anxiety disorders are more common among patients with thyroid disorders [13]. Patients with DTC may suffer SCH symptoms, which may impair QoL [14,15]. The aim of the present study is to investigate the effects of exogenous SCH during the follow-up of differentiated thyroid cancers on patients’ QoL and symptoms of anxiety-depression.

## 2. Materials and methods

### 2.1. Study population

A total of 278 patients who underwent thyroid surgery and admitted to Başkent University Ankara Hospital, Endocrinology outpatient clinic between 01/2015–01/2016 were included in the study. The patients were divided into exogenous SCH by LT4-DTC (n = 127), euthyroid-DTC (n = 66) and exogenous euthyroid-BTN who underwent thyroidectomy for benign thyroid pathology (n = 85) groups. SF-36 quality of life survey and Beck anxiety-depression inventory were performed on patients. Patients aged 18 years or above, who are educated enough to appropriately fill the self-evaluation form on their own, with no previous history of psychiatric disorder or substance abuse were included in the study. Free T3, fT4 and TSH levels were measured and recorded from the patients’ fasting blood samples, before the administration of LT4. Serum TSH (0.48–4.94 mU/L), fT4 (0.7–1.48 ng/dL), fT3 (1.71–3.71 ng/L) levels were measured using electro-chemiluminescence immunoassay (ECLIA) method. Subclinical hyperthyroidism was defined as having serum TSH levels below the reference range, and fT3 and fT4 levels within the reference range [2].

### 2.2. Instruments 

#### 2.2.1. Beck depression inventory (BDI)/Beck anxiety inventory (BAI)

This inventory is a self-assessment scale for rating a patient’s level and severity of depression/anxiety. It aims to rate the severity of depressive symptoms. It consists of 21 items, each of which is scored between 0–3 points. A high total point indicates a more severe depression/anxiety. A score of 0–13 is considered normal; 14–19 points indicate mild depression; 20–28 points indicate moderate depression, and 29–63 points indicate severe depression [16]. As for BAI; 0–7 points are considered normal; 8–15 points indicate mild anxiety; 16–25 points indicate moderate anxiety; and 26–63 points indicate severe anxiety [17]. The reliability and validity of the Turkish versions of these instruments have been examined [18,19].

#### 2.2.2. Health survey short form-36 (SF-36)

The SF-36 is used to assess QoL [20]. This instrument is a 36-item self-report questionnaire with eight scales measuring major domains of functional health and well-being, role limitations due to physical problems, body pain, general health, vitality, social functioning, role limitations due to emotional problems and mental health. Each scale provides a score ranging from 0 to 100, with higher scores indicating better health status. The validity and reliability of the Turkish versions of SF-36 have been extensively documented [21].

### 2.3. Data analysis

For this study’s power analysis, G-power 3.1 softwares were used. In the study, 3 independent groups were compared. Power test F test ANOVA one-way was used. In the study, effect size 0.24 Type 1 error was taken 0.05 and the power of the study is 95%. All data analyses were performed with SPSS software (Statistical Package for the Social Sciences, version 15.0). Normality distribution analysis of the data was performed by using Kolmogorov-Smirnov and Shapiro-Wilk tests. One-way ANOVA analysis of variance and Tukey’s multiple comparison posthoc tests were used for comparison of qualitative variables among groups. The differences between independent groups in terms of numerical variables were examined using the Student’s t- test to compare means. Chi-square (χ2) test was used to analyze categorical variables. Pearson’s tests were used to test correlations and the degree of associations among variables. A P-value of < 0.05 was considered statistically significant.

## 3. Results

The groups were similar in terms of the mean age and sex distribution of patients (P = 0.68 P = 0.3). Free T4 level was significantly higher in SCH-DTC group than euthyroid-BTN group (P = 0.002), and was similar to the level in euthyroid-DTC group (P = 0.17) (Table 1). 

**Table 1:** General characteristics of the study groups.

Characteristics	SCH-DTCn = 127	Euthyroid-DTCn = 66	Euthyroid-BTNn = 85	P	P*	P**	P***
Age ± SD	51.92 ± 11.82	51.62 ± 14.06	50.1 ± 12.17	0.68			
Sex				0.3			
Female n (%)	106 (83.5)	53 (80.3)	61 (71.8)				
Male n (%)	21 (16.5)	13 (19.7)	24 (28.2)				
TSH mU/L± SD	0.12 ± 0.12	1.14 ± 0.73	1.9 ± 1.27		<0.001	<0.001	<0.001
fT3 ng/L± SD	3.1 ± 0.26	2.8 ± 0.35	2.87 ± 0.35		0.051	0.13	0.66
fT4 ng/dL ± SD	1.33 ± 0.22	1.24 ± 0.14	1.12 ± 0.13		0.17	0.002	0.06

DTC: Differentiated thyroid cancer, BTN: Benign thyroid nodule, fT3: Free triiodothyronine, fT4: Free thyroxine, SCH: Subclinical hyperthyroidism, TSH: Thyroid stimulating hormone, SD: Standard deviation, P: Difference between all groups, P*: Difference between SCH-DTC and euthyroid-DTC, P**: Difference between SCH-DTC and euthyroid-BTN, P***: Difference between euthyroid-DTC and euthyroid-BTN, P-value <0.05 was considered statistically significant, Age P: Difference between SCH-DTC, euthyroid-DTC and euthyroid-BTN (ANOVA test), Sex P: Difference between SCH-DTC, euthyroid-DTC and euthyroid-BTN (Chi-square test), p* p** p*** : Student’s t- test.

When groups were compared in terms of BDI, it was found that 72.4% (n = 92) of the patients in SCH-DTC group were normal, whereas 16.5% (n = 21) had mild, 8.7% (n = 11) had moderate, and 2.4% (n = 3) had severe depression. In euthyroid-DTC group, 83.3% (n = 55) of the patients were normal, whereas 9.1% (n = 6) had mild, 6.1% (n = 4) had moderate, and 1.5% (n = 1) had severe depression. In euthyroid-BTN group, 87.1% (n = 74) of the patients were normal, and 10.6% (n = 9) had mild, 1.2% (n = 1) had moderate, and 1.2% (n = 1) had severe depression, and the groups were similar in terms of rates of depression severity (P = 0.15) (Table 2). When groups were compared in terms of BAI, it was found that 32.3% (n = 41) of the patients in SCH-DTC were normal, whereas 40.2% (n = 51) had mild, 15.7% (n = 20) had moderate, and 11.8% (n = 15) had severe anxiety. In euthyroid-DTC group, 57.6% (n = 38) of the patients were normal, and 24.2% (n = 16) had mild, 13.6% (n = 9) had moderate and 4.5% (n = 3) had severe anxiety. In euthyroid-BTN group, 48.2% (n = 41) of the patients were normal, and 42.4% (n = 36) had mild, 9.4% (n = 8) had moderate anxiety and severe anxiety was not detected in any of the patients. The rate of moderate/severe anxiety in SCH-DTC group was 27.5% (n = 35), which was significantly higher than euthyroid-BTN group with 9.4% (n = 8) (P = 0.001) (Table 2). 

**Table 2:** Comparison of groups in terms of Beck depression and anxiety scores.

	SCH-DTCn = 127	Euthyroid-DTCn = 66	Euthyroid-BTNn = 85	P	P*	P**	P***
Beck depression score				0.15			
Normal (0–13) n (%)	92 (72.4)	55 (83.3)	74 (87.1)				
Mild (14–19) n (%)	21 (16.5)	6 (9.1)	9 (10.6)				
Moderate (20–28) n (%)	11 (8.7)	4 (6.1)	1 (1.2)				
Severe (29–63) n (%)	3 (2.4)	1 (1.5)	1 (1.2)				
							
Beck anxiety score				0.001			
Normal (0–7) n (%)	41 (32.3)	38 (57.6)	41 (48.2)	0.002	0.001	0.01	0.25
Mild (8–15) n (%)	51 (40.2)	16 (24.2)	36 (42.4)	0.03	0.028	0.75	0.02
Moderate (16–25) n (%)	20 (15.7)	9 (13.6)	8 (9.4)	0.41			
Severe (26–63) n (%)	15 (11.8)	3 (4.5)	0 (0)	0.002	0.1	0.001	0.08
Moderate + Severe n (%)	35 (27.5)	12 (18.1)	8 (9.4)	0.005	0.15	0.001	0.11

DTC: Differentiated thyroid cancer, BTN: Benign thyroid nodule, SCH: Subclinical hyperthyroidism, P: Difference between SCH-DTC, euthyroid-DTC and euthyroid- BTN (Chi-square test), P*: Difference between SCH-DTC and euthyroid-DTC, P**: Difference between SCH-DTC and euthyroid-BTN, P***: Difference between euthyroid-DTC and euthyroid- BTN, P-value <0.05 was considered statistically significant.

In terms of SF-36 quality of life survey, SCH- DTC group scored lower than euthyroid-BTN group in all scales of the survey (physical functioning, role limitation due to physical problems, body pain, general health, vitality, social functioning, role limitation due to emotional problems, mental health) (P < 0.001, P = 0.03, P = 0.002, P < 0.001, P < 0.001, P < 0.001, P < 0.001, P < 0.001). Vitality and mental health scores were significantly lower in SCH-DTC group than euthyroid-DTC group (P = 0.007, P = 0.03, respectively) (Table 3). 

**Table 3:** Comparison of groups in terms of SF-36 scale scores.

	SCH-DTC n = 127	Euthyroid-DTCn = 66	Euthyroid-BTNn = 85	P	P*	P**	P***
SF-36 scale (mean ± SD)							
Physical functioning	73.54 ± 22.98	81.97 ± 44.96	83 ± 15.85	0.02	0.08	<0.001	0.17
Role- physical	71.65 ± 52.55	74.62 ± 37.09	82.94 ± 25.64	0.04	0.68	0.03	0.37
Body pain	67.87 ± 22.87	73.33 ± 20.48	76.7 ± 18.4	0.02	0.1	0.002	0.3
General health	54.88 ± 19.55	60.53 ± 20.96	66.23 ± 15.48	<0.001	0.65	<0.001	0.12
Vitality	55.11 ± 18.79	62.57 ± 22.70	70 ± 15.1	<0.001	0.007	<0.001	0.052
Social functioning	72.05 ± 21.67	75.56 ± 22.19	86.4 ± 15.81	<0.001	0.29	<0.001	0.001
Role-emotional	60.62 ± 64.16	68.18 ± 41.52	86.66 ± 22.53	<0.001	0.38	<0.001	0.035
Mental health	60.03 ± 17.4	65.94 ± 19.16	71.76 ± 13.21	<0.001	0.03	<0.001	0.085
							

DTC: Differentiated thyroid cancer, BTN: Benign thyroid nodule, SCH: Subclinical hyperthyroidism, SD: Standard deviation, P: Difference between SCH-DTC, euthyroid-DTC and euthyroid-BTN (ANOVA test), P*: Difference between SCH-DTC and euthyroid-DTC, P**: Difference between SCH-DTC and euthyroid- BTN, P***: Difference between euthyroid-DTC and euthyroid-BTN, P-value <0.05 was considered statistically significant, P* P** P*** : Student’s t- test.

In the correlation analysis, no significant correlation was detected between TSH levels and BDI scores (P = 0.073 r = –0.1) . A significant negative correlation was detected between TSH levels and BAI scores (P = 0.009 r = –0.16). In terms of SF-36 quality of life survey, a statistically significant weak positive correlation was detected between TSH and physical functioning, body pain, general health, vitality, social functioning and mental health scales (P = 0.015 r = 0.14, P = 0.006 r= 0.16, P = 0.002 r = 0.18, P = 0.001 r =0.2, P = 0.01 r = 0.15, P = 0.001 r = 0.2, respectively). No correlation was detected between role limitation due to physical and emotional problems scales and TSH (P = 0.2 r = 0.07, P = 0.1 r = 0.1, respectively). A significant negative correlation was detected between fT4 and vitality, social functioning and mental health scales of SF-36 quality of life survey (P = 0.027 r = –0.42, P = 0.04 r = –0.4, P = 0.01 r = –0.48). A significant positive correlation was detected between fT4 and BAI (P = 0.04 r =0.4). There was no significant correlation between fT3 and SF36, BDI and BAI socres (Table 4). 

**Table 4:** Correlation between thyroid function tests and SF-36, BDI and BAI scales.

	TSHr	P	fT4r	P	fT3r	P
SF-36						
Physical functioning	0.14	0.015	–0.08	0.69	0.2	0.31
Role- physical	0.07	0.2	–0.08	0.68	0.05	0.8
Body pain	0.16	0.006	0.19	0.35	0.27	0.16
General health	0.18	0.002	–0.01	0.95	–0.21	0.27
Vitality	0.2	0.001	–0.42	0.027	–0.01	0.95
Social functioning	0.15	0.01	–0.4	0.04	–0.11	0.57
Role-emotional	0.1	0.1	–0.3	0.13	–0.26	0.18
Mental health	0.2	0.001	–0.48	0.01	–0.2	0.34
BDI	–0.1	0.07	0.3	0.11	0.1	0.6
BAI	–0.16	0.009	0.4	0.04	0.2	0.31

BAI: Beck anxiety inventory, BDI: Beck depression inventory, fT3: Free triiodothyronine, fT4: Free thyroxine, SCH: Subclinical hyperthyroidism, TSH: Thyroid stimulating hormone, P-value <0.05 was considered statistically significant.

## 4. Discussion

In our study, SCH-DTC group scored lower than euthyroid-BTN in most of the SF-36 QoL scores and lower than euthyroid-DTC in some of the SF-36 QoL scores. Among all malignancies, DTC has one of the highest remission rates and is associated with a high long-term survival rate [2]. The conventional treatment of DTC involves LT4 replacement and TSH suppression to minimize disease recurrence. However, the resulting subclinical hypothyroidism is associated with physical and psychological abnormalities that may adversely affect QoL [4,7]. In some cancer patients, being aware of the cancer diagnosis may also negatively affect their quality of life [22]. Therefore, diagnosing additional pathological conditions further impairing patients’ quality of life may benefit survival. Vigario et al. reported that patients with SCH-DTC undergoing LT4 suppression treatment had a worse quality of life compared to those with euthyroid DTC [23]. Similarly, De Oliveira Chachamovitz et al. compared 38 patients with SCH-DTC and 54 patients with euthyroid DTC, and showed that patients with SCH-DTC had a lower quality of life than patients with euthyroid DTC [7]. In these studies, fT4 levels in patients with SCH were significantly higher than the euthyroid group. In our study, since there were no difference between SCH-DTC and euthyroid-DTC groups in terms of fT4 levels, most of their quality of life scale scores were similar. Moreover, since fT4 level in SCH-DTC group was higher than euthyroid-BTN group, most of the quality of life scale scores were lower. It appears that quality of life is essentially affected by fT4 levels. 

Thyroid dysfunction is known to cause significant mental symptoms and depression anxiety disorders are more common among patients with thyroid disorders [13]. Subclinical hyperthyroidism may cause affect disorders [24]. Panic attack and generalized anxiety are common disorders in thyroid dysfunction, particularly in overt hyperthyroidism [13,25–28]. However, data on whether SCH increases anxiety are limited and contradictory [29,30]. Thyroid hormones affect neuropsychiatric condition by increasing the activity of catecholamines. Studies have shown that thyroid hormones increases the number and activity of beta-catecholamine receptors [31,32]. Sait Gönen et al. reported significantly higher anxiety scoresand fT4 levels in patients with SCH compared to an euthyroid population [11]. Correlation analysis of our study showed a weak albeit significant correlation between TSH and fT4 levels and BAI scores. We found a significantly higher rate of moderate/severe anxiety in the SCH group (27.5%) than euthyroid-BTN. Here, the determining factor is the difference between fT4 levels. Subclinical hypothyroid-DTC and euthyroid-DTC groups, which had similar fT4 levels, were also similar in terms of anxiety severity. It was reported that even a minimal change in thyroid hormones can have behavioral effects [33]. Based on these data, it can be said that anxiety severity is affected by fT4 levels in SCH. 

There is a small body of prospective data on the impact of SCH on depression [13]. Furthermore, studies with large patient series have yielded conflicting results [10,34]. In a 1219-patient study, Renate et al. concluded that patients with SCH had similar physical, cognitive functions and depression symptoms as euthyroid patients [34]. In a study of 1503 patients by Medici M et al., those with SCH had higher depressive symptom scores, and a prospective evaluation revealed that low TSH levels were associated with an increased incidence of depression [10]. The 3-year outcomes of another study showed that patients with SCH had higher depression scores than euthyroid ones [9]. Eustatia-Rutten et al. studied patients with DTC undergoing TSH suppression and showed that exogenously induced SCH was associated with higher depression scores than the euthyroid healthy control group [35]. Our study revealed similar rates of severe depression among SCH and control groups. TSH and BDI scores were not correlated. These differences may be due to the absence of the inclusion of multiple other factors affecting the results.

In conclusion; TSH suppression therapy administered for differentiated thyroid cancers are associated with significantly increased anxiety symptom scores but not depression scores. Most parameters of the QoL rating scale were negatively affected by TSH suppression. According to our findings, fT4 levels are correlated with anxiety in SCH-DTC, and it may have therefore caused disruption in the quality of life. It must be kept in mind that SCH induced by TSH suppression in patients with DTC can disrupt the patients’ psychological status and quality of life.

## Acknowledgments/Disclaimers

The authors would like to thank the Department of Endocrinology and Metabolism and the Department of Biochemistry (Başkent University Faculty of Medicine) for the laboratory work. This study did not receive any financial funding and was performed as part of the employment duties of the authors. This study has been approved by Başkent University of Medical Sciences Ethics Committee and was performed in accordance with the ethical standards laid down in the 1964 Declaration of Helsinki and its later amendments. The present study protocol was reviewed and approved by the Institutional Review Board of Başkent University College of Medicine (Approval no. KA14/314).

## Conflict of interest

All of the authors declare that they have no competing interests. The authors also certify that there is no conflict of interest with any financial organization regarding the material discussed in the manuscript. Authors are those who have contributed to the conception and design of the article, the acquisition of data, or the analysis and interpretation of data, as well as the writing of the article or the revision of its content; and have read and approved the final version of the article before submission.
